# The Impact of Taste Preference-Related Gene Polymorphisms on Alcohol Consumption Behavior: A Systematic Review

**DOI:** 10.3390/ijms232415989

**Published:** 2022-12-15

**Authors:** Ali Abbas Mohammad Kurshed, Róza Ádány, Judit Diószegi

**Affiliations:** 1 Department of Public Health and Epidemiology, Faculty of Medicine, University of Debrecen, Kassai Street 26/B, H-4028 Debrecen, Hungary; 2 Doctoral School of Health Sciences, University of Debrecen, Nagyerdei Krt. 98., H-4032 Debrecen, Hungary; 3 ELKH-DE Public Health Research Group, Department of Public Health and Epidemiology, Faculty of Medicine, Kassai Street 26/B, H-4028 Debrecen, Hungary

**Keywords:** alcohol consumption, genetics, polymorphism, taste preference, taste sensitivity

## Abstract

Unhealthy alcohol consumption is recognized as a leading contributory factor to mortality and disability. In addition to other factors, taste sensation also mediates alcohol intake. The orosensation provoked by alcoholic drinks may vary across individuals and may be responsible for differences in preference for alcoholic beverages. Thus, individual genetic variability of taste preference may have an impact on alcohol consumption practices. The present review aimed to explore the associations between different taste preference polymorphisms and alcohol consumption behavior. Based on the PRISMA statement, the three databases PubMed, Web of Science and ProQuest Central were searched to identify articles and the Q-Genie tool was used to assess the quality of the included studies. Among the 17 studies included in this review, 5 and 12 were of good and moderate quality, respectively. Most of the studies analyzed TAS2R38 (taste 2 receptor member 38) rs713598, rs1726866, rs10246939 polymorphisms. Due to the inconclusive findings on these variants and the very limited number of studies on other polymorphisms, additional extensive research is recommended to replicate the existing findings, to generate new knowledge to enhance our understanding of the complexity of alcohol consumption behavior and to aid the development of personalized recommendations on unhealthy alcohol use.

## 1. Introduction

Consumption of alcohol became widespread throughout the world due to industrialization of production, globalization of marketing and promotion of alcohol. Although the findings of some studies indicated that intake of low amounts of alcohol is beneficial to decrease the incidence of a few non-communicable chronic diseases such as type 2 diabetes [1] and cardiovascular diseases [2], the harmful effects of alcohol on disease and injury outweighed these positive effects. Heavy alcohol consumption is related to an enhanced risk for numerous negative health consequences comprising all-cause mortality, cancer, cardiovascular diseases and injuries [3,4,5,6]. The World Health Organization (WHO) Global status report on alcohol and health demonstrated that alcohol consumption is linked with more than 200 illnesses and/or health-related disorders, among which the most common and predominant ones include alcohol use disorders, liver cirrhosis, cancers and injuries [7]. In addition, it was also articulated from an analysis that daily consumption of zero standard drinks decreased the risk of all overall health loss, while the risk rose monotonically due to higher amounts of drinking [3]. In 2020 harmful alcohol consumption accounted for 1.78 million deaths worldwide [8]. Moreover, alcohol use was identified as the leading risk factor (at level 4) in the 25–49 years age group [9]. In addition to the individual physical and mental health problems caused by alcohol use, socioeconomic consequences for the drinker and harm to others is also a substantial problem [7].

Both the amount of alcohol consumed and patterns of drinking involving heavy drinking during special occasions are responsible for the negative health consequences of alcohol intake [10,11]. Consumption as well as drinking patterns of alcohol are influenced by a variety of factors encompassing environmental (income, occupation, sex, education and psychological) and genetic factors [12,13,14]. In addition, sensory and olfactory components of flavor and temperature of alcohol are also considered to be influential factors of alcohol consumption [15]. Perceived taste is one of the many sensory components thought to determine alcohol intake. Studies indicate that alcohol preference and intake may be influenced by sensitivity to bitter and sweet taste modalities, although the findings are somewhat equivocal as reviewed by Thibodeau and Pickering [16], which may be due to methodological differences in characterizing alcohol consumption. It may be hypothesized that higher sensitivity to bitter-perceived compounds (quinine, phenylthiocarbamide-PTC and 6-n-propylthiouracil-PROP) and higher detection threshold for sucrose and higher preferences for sweetness may be linked to an increased risk of alcohol-related problems, to which the latter finding may be more prominent among men compared to women [16].

The development of alcohol use disorder is multifactorial, encompassing both environmental and genetic factors [17] with heritability estimates ranging from 50 to 60 per cent [18,19]. The most extensively studied genetic variants regarding alcohol use disorder (AUD) and alcohol consumption are involved in the pathway of ethanol metabolism (alcohol dehydrogenase—ADH; aldehyde dehydrogenase—ALDH) and are related to neurotransmitters mediating the positive reinforcing effects of alcohol [20]. Although less extensively studied, taste preference gene polymorphisms may also mediate alcohol consumption behaviors. Numerous genes are responsible for mediating taste preferences, including alcohol preference [21]. An individual’s preference for specific tastes can determine the choice of alcoholic beverages and thus eventually influence both the amount and pattern of alcohol consumption. This systematic review aimed to elucidate the association between genetic polymorphisms related to taste preference modalities and various aspects of drinking behaviors. Knowledge gathered through this review can be one of the stimulating factors in the development of public policy to address alcohol use disorders and thereby mitigate disease burden originating from unhealthy practices of alcohol consumption.

## 2. Results and Discussion

### 2.1. Search Outcomes

**Figure 1:**
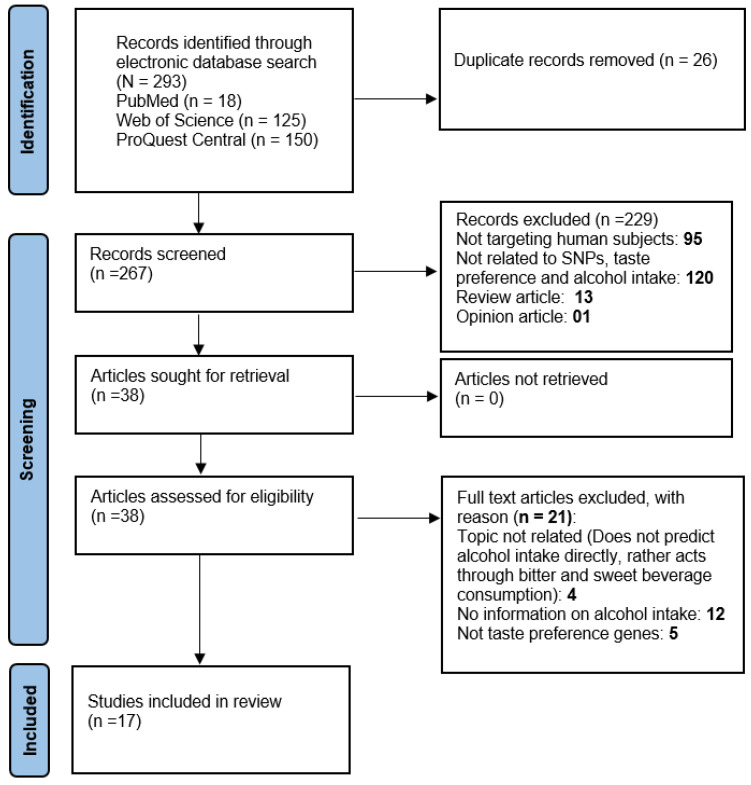
PRISMA flowchart of study selection process [22].

Altogether 293 studies were identified, 18 in PubMed, 125 in Web of Science and 150 in ProQuest Central. After removing duplicates (n = 26), abstracts of the remaining articles (n = 267) were screened individually by two authors (AAMK, JD). Studies unable to meet the inclusion criteria (n = 229) were excluded from further analysis, resulting in 38 articles for full-text assessment to check eligibility for subsequent analysis. Due to specific reasons (presented in Figure 1) 21 articles were excluded from the study. Finally, 17 publications were included in this review for in-depth analysis and interpretation.

### 2.2. Studies Included in the Review

Included studies of this review focused on 43 single nucleotide polymorphisms (SNPs) of 27 different taste preference-related genes (description of the single nucleotide polymorphisms can be found in Table 1). The relationships between different dimensions of alcohol consumption behavior and TAS2R38 rs713598 (n = 10), rs1726866 (n = 9), rs10246939 (n = 8) followed by TAS2R16 (Taste 2 Receptor Member 16) rs846672 (n = 4); TAS2R16 rs1308724 (n = 3); TAS2R50 (Taste 2 Receptor Member 50) rs1376251 (n = 3); TAS2R16 rs846664 (n = 3); TAS2R19 (Taste 2 Receptor Member 19) rs10772420 (n = 3); TAS2R20 (Taste 2 Receptor Member 19) rs12226920 (n = 2); TAS2R8 rs1548803 (n = 2); and CA6 (Carbonic Anhydrase 6) rs2274333 (n = 2) were demonstrated by multiple studies. The effects of other included polymorphisms on alcohol intake patterns were examined only by single studies.

### 2.3. Quality Assessment of Included Studies

Among the 17 included articles, 5 (29.4%) were rated good quality, while 12 (70.6%) of them were of moderate quality.

### 2.4. Characteristics of Studies Included in the Review

To explore the interaction between TAS2R38 genotype and alcohol intake, a study was conducted by Beckett et al. upon 180 18–88 year old hospital patients (Gosford Hospital, NSW) in Australia, who provided information on alcohol consumption using a Food Frequency Questionnaire (FFQ). Alcohol consumption was converted to standard drinks (1 standard drinks = 10 mL of alcohol). Study results showed that TAS2R38 P49A genotype was associated with daily consumption of standard drinks while no association was found with the consumption of alcohol in beer and wine [56]. The study performed by Hayes on 96 healthy adults from a rural college campus of the University of Connecticut community, who did not smoke more than nine cigarettes weekly, revealed that TAS2R16 rs846672 was significantly associated with frequency of alcoholic beverage consumption, and carriers of CC homozygotes of TAS2R16 rs1308724 consumed alcohol less frequently than heterozygotes. In this study a semiquantitative food frequency survey was applied to quantify (total quantity and frequency–number of times per year) beer, wine, and liquor consumption, where standard drinks equaled 12, 5, and 1.5 oz., respectively [38]. Ramos-Lopez and co-authors presented that among 375 non-smoker Mestizo individuals without prescribed medication and chronic sinus problems, TAS2R38 AVV/AVV genotype was significantly associated with alcohol intake. Alcohol intake was assessed by a medical history questionnaire and expressed as: g ethanol = volume mL × % alcohol × 0.8/100. (1 shot of tequila = 35 mL (11.2 g ethanol); 1 beer = 330 mL (12.3 g ethanol); and 1 glass of red wine = 120 mL (14.4 g ethanol)). Furthermore, participants were classified as drinkers (DRS) and nondrinkers (NDRS), where DRS consumed more than two drinks per occasion and NDRS consumed equal or less than two drinks per occasion [57]. Duffy et al. depicted that among 86 healthy non-smoker adults of primarily European ancestry without dietary restraint and no uncommon haplotype carriers of TAS2R38, AVI/AVI homozygotes drank significantly more alcohol compared to PAV/AVI heterozygotes or PAV/PAV homozygotes. Version 98.1 of the Block Food Survey was used to measure yearly intake of beer, wine or wine coolers, liquors or mixed drinks, where ranges from “never” to “every day” were used for consumption frequency, and glass, bottle, drink and the size of the serving for amount of consumption per time interval [58]. Fu D. and colleagues conducted a study on 519 respondents of mostly European descent where alcohol consumption frequency was recorded as “<2 drinks per week”, “2–7 drinks per week” and “>7 drinks per week”, and the results showed that TAS2R38 variants (rs10246939, rs1726866, rs713598) had a significant association with alcohol consumption, while the relationship between CA6 rs2274333, GNAT3 rs1524600, TAS2R16 rs846664, TAS2R16 rs846672, TAS2R19 rs10772420, TAS2R20 rs12226920, TAS2R43 rs71443637, TAS2R46 rs2708377, TAS2R50 rs10772397, TAS2R60 rs4595035, TAS2R8 rs1548803 and frequency of alcohol consumption was not statistically significant [41]. It is articulated in the study of Hinrichs et al. that regardless of ethnicity, individuals with the ancestral allele of TAS2R16 rs846664 were found to be at increased risk of alcohol dependence, and TAS2R16 rs978739, rs860170, rs1204014 SNPs had no significant association with alcohol dependence when investigating 262 families. During this study alcohol dependence was assessed according to DSM-IIIR (Diagnostic and Statistical Manual of Mental Disorders, 3rd Edition Revised), DSM-IV (Diagnostic and Statistical Manual of Mental Disorders, 4th edition), Feighner criteria and ICD-10 (International Classification of diseases 10th Revision) criteria [35]. Wang and colleagues demonstrated that among participants of mostly European descent (262 families) TAS2R16 rs846664 was associated with lower alcohol dependence risk, and the common taster haplotype TAS2R38 was significantly associated with a lower mean of the largest number of drinks that participants had ever had in a 24 h period compared with the other haplotypes. The Semi-Structured Assessment for the Genetics of Alcoholism (SSAGA) tool was used to assess alcohol dependence, the largest number of drinks ever had in a 24 h period, age at first intoxication as well as age of onset of regular drinking (at least once a month for 6 months or more) [59]. Choi et al. conducted a study on 1524 Korean participants meeting the inclusion criteria (no diabetes mellitus, severe systemic or mental disease, history of any other cancer within the past five years or advanced gastric cancer, missing genotype, missing dietary data, total energy intake < 500 kcal or > 5000 kcal), where alcohol consumption (beer, hard liquor, Korean spirits, Korean rice wine, wine and fruit liquor) was assessed by a self-administered FFQ. The study was part of the gastric cancer research project at the National Cancer Center (NCC) Korea. The outcome of the study described that TAS2R38 diplotype was not associated with alcohol consumption [60]. Another study published by Choi and co-authors revealed that TAS2R38 AVI carriers were less likely to be drinkers, and TAS2R5 rs2227264 predicted total alcohol consumption. TAS1R3 rs307355 CT carriers were associated with heavy drinking status and the homo-recessive types of TAS2R4 rs2233998 and TAS2R5 rs2227264 were associated with rice wine consumption, and TAS1R2 rs35874116 was associated with wine drinking and consumption level. Furthermore, TAS2R50 rs1376251 was associated with rice wine and spirit consumption. Participants of this study were free of systemic or mental disorder symptoms, diabetes mellitus and history of any cancers within the past five years, and visited the National Cancer Center in Korea to obtain a health screening examination. Individuals were classified as current, past or never drinkers. The frequency of alcohol consumption (one, two to three or four to six times a day, week or month), the amount of alcoholic beverages consumed on a typical day when drinking (by glass) and the type of alcoholic beverage consumed (beer, Soju, spirits, rice wine, wine and other) were recorded for current and past drinkers (ever drinkers). Total daily alcohol consumption (g/day) was estimated from the frequency of drinking, the amount of alcohol consumed (mL) and the ethanol content of drinks consumed. Drinkers consuming more than 30 g/day of alcohol were defined as heavy drinkers, and subjects were considered as light drinkers if they consumed <30 g/day [26]. Keller et al. revealed that among 1007 German participants without type 2 diabetes, lower alcohol intake per week was observed in the PAV group [61]. Vinuthalakshmi et al. used a questionnaire on lifestyle habits and demonstrated that a positive significant association was found between the TAS2R38 AVI/AVI haplotype and alcoholism among 296 subjects from the Koraga primitive tribes of India, where the participants were in good health and free from sinus problem and food allergies. Additionally, pregnant and/or lactating women and subjects taking medications that might influence sensory perception were also excluded from the study [62]. Another study included in this review conducted by Dotson and co-researchers used the first three questions of the AUDIT (Alcohol Use Disorder Identification Test) screening tool to assess alcohol consumption patterns. It was conducted on 173 head and neck cancer patients from clinics at the University of Florida (94% white participants). In this study TAS2R38 was significantly associated with the first question of the AUDIT, while rs1015443 of TAS2R13 was associated with the second and third questions of the AUDIT tool. In addition, no association was found for TAS2R3 rs765007, TAS2R4 rs2234001, TAS2R5 rs2234012, TAS2R38 rs10246939, TAS2R38 rs1726866, TAS2R39 rs4726600, TAS2R40 rs10260248, TAS2R41 rs1404635, TAS2R7 rs619381, TAS2R8 rs1548803, TAS2R10 rs10845219, TAS2R14 rs7138535, TAS2R14 rs1376251, TAS2R20 rs10845281, TAS2R19 rs10772420 and PRH1-TAS2R14 rs11612527 variants [28]. Choi et al. depicted that there was no evidence of associations between TAS2R38 and alcohol intake. In this study 3567 participants were included from three rural areas (Goryeong, Namwon and Yangpyeong) of Korea, where a structured questionnaire was used to collect data. Individuals were classified as never, past or current drinkers [63]. In 2017 Choi et al. investigated daily alcohol consumption (g/day) of 2042 individuals from the National Cancer Center Korea and found no associations between TAS2R38 (rs713598, rs1726866, rs10246939) PAV and AVI haplotypes or CA6 rs2274333 and alcohol consumption either individually or combined [64]. Timpson and his colleagues reported no association between TAS2R38 and alcohol consumption among 3383 British women randomly selected from 23 British towns. To assess alcohol consumption this research applied six alcohol consumption categories: never, on special occasions, once or twice a month, weekends only, most days and daily. Then, these six categories were merged into two categories: consumption of alcohol at any frequency and no alcohol consumption [65]. The outcome of Schembre’s study showed that among subjects of Japanese American, white or Native Hawaiian ancestry, TAS2R38 PAV/PAV diplotype, TAS2R50 rs1376251 and the TAS2R16 rs846672 polymorphism had no significant effect on alcohol intake. Study participants were 914 colorectal adenoma cases and 1188 controls. Alcohol consumption (including beer, wine, hard liquor and other alcohol) was assessed by an FFQ [42]. Furthermore, Ong et al. presented that among 438 870 individuals of white British ancestry TAS2R38 rs1726866 was inversely associated with alcohol consumption, but for TAS2R19 rs10772420 and PRH1-TAS2R14 rs2597979 the association was not statistically significant. In this study drinking frequency (6-point frequency scale ranging from never to daily) was assessed (non-drinkers = no consumption of alcohol, while heavy drinkers = more frequently than 3–4 times weekly) [53]. Summary of included studies in this review is depicted in Table 2.

### 2.5. Phenotype Assessment Methods Used in the Studies Reviewed

Various phenotype assessment methods were applied in the studies included in this review. Most of the phenotyping approaches used a food frequency questionnaire [42,56,60] or other questionnaires [26,38,41,53,57,58,61,63,64] to obtain information on alcohol consumption behavior; however, different outcomes on phenotypes were analyzed, i.e., daily consumption (standard drinks [56], g/milligram/day [26,42,57,60,63,65]), weekly consumption [61], yearly consumption [58] and consumption frequency (per year [38], per week [41]), and some researchers also categorized enrolled subjects as drinkers, nondrinkers [26,57,64] and heavy drinkers [26,53] based on their drinking patterns. Furthermore, some studies investigated the association between genetic polymorphisms and the consumption of different types of alcoholic beverages, not only total alcohol consumption [26,56]. In addition, Dotson et al. applied the first three questions of the AUDIT [28] and others identified alcohol dependent individuals by different criteria (ICD-10, DSM-IIIR, DSM-IV and Feighner criteria [35], Semi-Structured Assessment for the Genetics of Alcoholism [59] and by a questionnaire) [62].

### 2.6. Effect of Genetic Polymorphisms on Alcohol Consumption

The three functional polymorphisms rs713598, rs1726866 and rs10246939 of TAS2R38 are responsible for variability in human bitter taste perception and preference, and sweet preference as well [21]. These variants were the most extensively studied in terms of alcohol consumption behavior among the studies included in the review [26,41,53,56,59,62]. A significant association was found between three tightly linked variants of TAS2R38 (rs10246939, rs1726866 and rs713598) and alcohol consumption frequency [41]. Carriers of the alleles allowing perception of bitterness in PTC consumed alcohol at higher frequencies [41]. Additionally, AVI/AVI homozygotes drank alcohol more frequently and consumed significantly more alcoholic beverages than individuals with other genotypes [38,58]. Furthermore, the common taster haplotype (PAV) was significantly associated with a lower mean of the largest number of drinks that participants ever had in a 24 h period compared with other haplotypes [59]. Moreover, the dominant model analyses (PAV/PAV vs. PAV/AVI+AVI/AVI) confirmed that the subjects with the AVI haplotype were less likely to be drinkers [26,61], while contrasting findings from other studies stated that a positive association existed between the non-taster haplotype AVI/AVI and alcoholism [62], and carriers of at least one PAV allele show significantly lower alcohol intake [61]. In another study from Mexico (city of Guadalajara; state of Jalisco) the frequency of AVV homozygotes was significantly higher among drinkers and was also associated with increased alcohol intake compared to heterozygotes and PAI homozygotes [57]. On the contrary, one study conducted among Korean males and females demonstrated that TAS2R38 diplotypes (PAV/PAV, PAV/AVI and AVI/AVI) showed no significant differences in daily alcohol consumption [60]. A study conducted on head and neck cancer patients described that the C allele of TAS2R38 rs713598 variant was strongly associated with decreased alcohol consumption [28]. In addition, this variant was a significant predictor of the number of standard drinks consumed. Subjects carrying the p allele consumed fewer standard drinks per day from spirits as well as from mixed drinks, compared to non-carriers [56]. Likewise, TAS2R38 rs1726866 had an inverse significant association with alcohol consumption frequency and drinker status [53]. In contrast, the TAS2R38 rs713598 variant showed no significant association with the second and third questions of the AUDIT screening tool [28], beer and wine consumption [56] and alcohol intake per week [61], while variants TAS2R38 rs1726866 and TAS2R38 rs10246939 showed no significant association with the first three questions of the AUDIT screening tool [28] and alcohol intake per week [61]. On the other hand, other studies revealed no association between the three SNPs rs10246939, rs1726866 and rs713598 of TAS2R38 with beer and total daily alcohol consumption [26,65], alcohol drinker status [63,64], frequency and amount of alcohol consumed [42].

Regarding the other polymorphisms, which were investigated by several studies, it was demonstrated that AA carriers of TAS2R16 rs846672 consumed alcoholic beverages twice as frequently as the heterozygotes or major allele homozygotes and also drank more than G allele carriers [38], while TAS2R16 rs1308724 CC homozygotes consumed alcohol less frequently than heterozygotes, who also consumed less frequently than GG homozygotes [38]. On the contrary other studies identified no association between these variants with different alcohol consumption behaviors [38,41,42].

Another variant of the TAS2R receptor family, TAS2R50 rs1376251 showed a significant association with drinker status of spirits, while participants with the CC genotype tended to drink more rice wine compared to other genotypes [26]. However, this polymorphism was not linked to daily alcohol intake (mg/day) [42], and neither to Soju, beer and wine consumption [26] in other studies. Additionally, the ancestral K172 allele of TAS2R16 rs846664 was associated with increased risk of alcohol dependence, regardless of ethnicity [35], while the alcohol dependence risk allele was associated with lower alcohol dependence risk and also associated with lower mean of the largest number of drinks that participants had ever had in a 24 h period in African American families [59]. Meanwhile, no significant association was observed between this genetic variant and frequency of alcohol consumption by Fu et al. [41]. The association between TAS2R19 rs10772420 and drinking behavior frequency [41,53], heavy drinker status [53] and with the first three questions of the AUDIT screening tool [28] was not statistically significant [53]. It was also demonstrated that the TAS2R20 variant rs12226920 had a significant association with drinker status of spirits [26], while there was no association with beer consumption [41]. Moreover, the polymorphism rs1548803 of TAS2R8 was also found not to be linked to alcohol consumption behavior as measured by the first three questions of the AUDIT screening tool [28], and with alcohol consumption frequency [41]. Similarly, as was shown in two independent studies, CA6 rs2274333 was not associated with daily alcohol consumption [65] and with alcohol consumption frequency [41]. Effects of taste preference gene polymorphisms on alcohol consumption, which were investigated by single studies, are detailed in Table 3.

### 2.7. Discussion

To our knowledge, this is the first systematic review aiming to explore and compile evidences regarding associations between different taste preference gene polymorphisms and various aspects of alcohol drinking behaviors. Previous systematic reviews and meta-analyses in the literature focus on the contribution of genes involved in different pathways of alcohol metabolism and various neurotransmitter systems contributing to the development of alcohol use disorder. Most of them examine single genes and polymorphisms (ADH1B rs122998 [66]; BDNF rs6265 [67]; GABRA2 rs279858 and rs567926 [68]; and OPRM1 rs1799971 [69]), or those genes which are the most extensively studied [70]. Besides the genetic polymorphisms affecting the metabolic pathways of alcohol degradation and neurotransmitter systems involved in the development of alcohol/substance use disorder, patterns of alcohol consumption may also be influenced by taste and related genetic variants. The identification of these genetic polymorphisms and the extent of their effect on drinking habits may be useful in understanding the background of excessive alcohol consumption. In this review, we provide an overview of the impact of taste preference gene polymorphisms on different aspects of alcohol consumption.

The majority of the studies of this review focused on TAS2R38 rs713598, rs1726866 and rs10246939 variants, whose polymorphisms define the bitter supertaster–taster–non-taster phenotypes well [71,72], and are highly linked to bitter taste preference; moreover, convincing results were also found related to sweet-taste sensitivity and preference [21]. Our findings represent that these variants show inconsistent associations with various aspects of drinking behavior. The majority of studies with positive findings exhibited that individuals with the AVI haplotype drank significantly more alcoholic beverages [58] and more frequently [57] than other genotypes and a positive association was identified with drinker status as well [62], and only one study providing contrasting results was published, namely subjects with the AVI haplotype were less likely to be drinkers compared to other genotypes [26]. Meanwhile, in a Mexican study with 375 subjects from the city of Guadalajara (state of Jalisco) AVV and PAI haplotypes dominated and an association was demonstrated between AVV homozygotes and drinker status and alcohol consumption [38]. Otherwise, similar associations were found when the effects of these polymorphisms were analyzed individually [41,53,56]. These three TAS2R38 functional SNPs (rs713598, rs1726866 and rs10246939) define the bitter supertaster–taster–non-taster categories. PAV (proline–alanine–valine) homozygotes determine the PROP/PTC taster category, AVI (alanine–valine–isoleucine) homozygotes are considered as non-tasters and heterozygotes are characterized by intermediate sensitivity [73,74]. Results of these studies are in line with the hypothesis that higher sensitivity to bitter-perceived compounds may be linked to an increased risk of alcohol-related problems [16].

On the other hand, numerous genetic association studies did not confirm these associations [26,28,42,56,60,61,62,63,64,65]. These contradictory results may be due to the taste profile of alcohol, which comprises both sweet and bitter precepts based on concentration, the type of alcoholic beverages and the quantity of sugar added [16]. In line with this, the lack of independent assessment of different types of alcoholic drinks in certain studies may also modify the results [16]. Environmental factors, such as religious and cultural norms can be contributory to the avoidance of alcoholic drinks [75,76,77], which may also lead to inconsistency in study findings. Furthermore, lack of replicability may be explained by small sample sizes, and it cannot be excluded that different alcohol-related phenotypes encompass various genetic backgrounds. In addition, underreporting of alcohol consumption in a survey has to be also considered when analyzing self-reported results [78].

The polymorphism rs846664 of the TAS2R16 was investigated by three research groups, and two identified associations with alcohol dependence and the largest quantity of drinks consumed during a 24 h period [42]. The derived allele of this nonsynonymous mutation has been shown to increase sensitivity to toxic b-glucopyranosides [34] and was linked to higher sensitivity to salicin, but not to PROP bitterness [21]. This variation leads to a functional change in the receptor [35]. The substitution is located in the extracellular loop 2 between transmembrane domains 4 and 5, whose domain is responsible for ligand binding [36,37]. This change may lead to altered taste-related signaling and bitter responsiveness potentially resulting in differences in alcohol preference/consumption. As a future direction, this variant could be a target of subsequent studies regarding alcohol consumption.

Although relationships were found previously with various bitter-tasting compounds [21] (quinine absinthin, amarogentin, cascarillin, grosheimin, quassin, PROP and unsweetened grapefruit juice), TAS2R19 rs10772420 (coding for an arginine-to-cysteine substitution at amino acid 299 (R299C)) [79] was found not to influence alcohol consumption behavior in our review [28,41,53]. Though, the effect of this missense mutation on more prominent quinine perception is also suggested to be attributed to strong linkage disequilibrium (LD) to other SNPs located in other TAS2R genes [43,79]. The CA6 rs2274333 SNP has also received much attention in recent research considering PROP phenotype, but yielded inconclusive findings [21]. This variant is responsible for an amino acid substitution in the protein sequence of carbonic anhydrase VI, which is associated with formation and function of fungiform papillae on the anterior tongue surface [80]. Hence, PROP sensitivity may be influenced by this polymorphism via acting on fungiform papilla development and maintenance [81]. Similarly to the aforementioned polymorphism of TAS2R19, no associations were confirmed related to this variant and alcohol consumption either [41,65]. While both these variants are related to bitter taste and may potentially influence alcohol consumption behavior, more studies are needed to be able to draw conclusions on the effect of these two polymorphisms on alcohol consumption phenotypes, and in the case of other bitter taste-related genetic polymorphisms as well.

As mentioned previously, some studies suggest that higher detection threshold for sucrose and higher preferences for sweetness may be linked to an increased risk of alcohol-related problems [16]. Therefore, it can be expected that variants related to sweet preferences would influence alcohol consumption. One study included in our review investigated the functional variant rs35874116 of TAS1R2, and it was found that CC and CT vs. TT was associated with higher intake of sweet foods [21]. The potential underlying mechanism of this effect is that the SNP located in the primary extracellular domain of the T1R2 is predicted to harbor the ligand-docking site for carbohydrates and sweet-tasting molecules [25], would contribute to differences in sweet taste perception, food and wine intake [26]. Regarding alcohol consumption, TAS1R2 rs35874116 exhibited significant association with wine intake, but for other alcoholic drinks such as beer, soju and spirits the association was not significant. Carriers of the C allele were less likely to be wine drinkers; however, CC genotype subjects were characterized by higher levels of consumption than TT genotypes, a discrepancy which could be attributed to the limited number of wine drinkers [26]. Furthermore, in the same study the TAS1R3 variant rs307355 CT carriers were more likely to be heavy drinkers, and a marginal association was identified with soju intake, but in the case of wine, beer and spirits, no association was identified [26]. Located in the 5′UTR region of TAS1R3, this cytosine to thymine substitution may affect gene transcription [27] and lead to changes in sweetness and alcohol perception [26]. Reduced taste sensitivity to sucrose was also identified in case of the T alleles of this variant [21]. In the absence of additional studies, further research is needed to elucidate the impact of these polymorphisms on alcohol consumption behavior, which is relevant for those polymorphisms as well, as they were only investigated by single studies.

Numerous limitations need to be taken into consideration while interpreting the results of this systematic review. Though a wide range of research is now being conducted on various aspects of taste preference genetics, the number of relevant studies for this review is limited, and numerous polymorphisms were investigated only by single studies without replicating the findings. Moreover, studies encompassed various alcohol phenotypes and used different criteria, and some polymorphisms were studied in small samples. Additionally, the sampling procedure, inclusion and exclusion criteria and composition of study subjects varied among the studies. Regardless of the aforementioned drawbacks, this review is the first designed to accumulate the findings of different original studies dealing with the associations of various taste preference genetic polymorphisms and alcohol consumption.

## 3. Materials and Methods

### 3.1. Search Strategy and Eligibility Criteria

Three electronic databases, PubMed, Web of Science and ProQuest Central were used for systematic search to identify articles relevant to our study objective. The search strategy followed the PRISMA statement [82]. The search terms encompassed both controlled terms such as MeSH in PubMed and free text terms and were based on the combination of the following keywords: (“taste preference” OR “taste perception” OR “taste sensitivity” OR “sweet taste preference” OR “sweet taste perception” OR “sweet taste sensitivity” OR “bitter taste preference” OR “bitter taste perception” OR “bitter taste sensitivity” OR “fat taste preference” OR “fat taste perception” OR “fat taste sensitivity” “salty taste preference” OR “salty taste perception” OR “salty taste sensitivity” OR “sour taste preference” OR “sour taste perception” OR “sour taste sensitivity” OR “umami taste preference” OR “umami taste perception” OR “umami taste sensitivity” OR “taste threshold” OR “sweet taste threshold” OR “fat taste threshold” OR “salty taste threshold” OR “sour taste threshold” OR “umami taste threshold”) AND (“genes”) AND (“alcohol consumption”) NOT (“animal”). The reference lists of the included articles were also checked for possible eligible publications.

Only the articles fulfilling the following criteria were included in this study: (i) written in English; (ii) published in peer-reviewed journal; (iii) is an original article; (iv) targeting only human subjects; and (v) available in a full-text format. The search was conducted on 25 September 2021.

Details of the article selection process for the present systematic review are depicted in Figure 1.

### 3.2. Quality Assessment of Included Studies

Two authors (AAMK, JD) checked the quality of the included studies independently using the validated Q-Genie tool. Strengthening the Reporting of Genetic Association Studies (STREGA) and Strengthening the Reporting of Genetic Risk Prediction Studies (GRIPS) were the basis of the development of this tool. Furthermore, recommendations from Diabetologia, Human Molecular Genetics and Nature Genetics, as well as guidelines of individual research groups also aided in the developmental process of this instrument [83]. This quality assessment tool of genetic association studies consists of 11 items which are formulated as questions to represent the following categories: (i) rationale for study, (ii) selection and definition of outcome of interest, (iii) selection and comparability of comparison groups, (iv) technical classification of the exposure, (v) non-technical classification of the exposure, (vi) other source of bias, (vii) sample size and power, (viii) a priori planning of analysis, (ix) statistical methods and control for confounding, (x) testing of assumptions and inferences for genetic analysis, and (xi) appropriateness of inferences drawn from results. Each item is scored on a 7-point Likert Scale ranging from 1 (poor) to 7 (excellent). Through reading and examining the included studies an overall quality score was also generated separately by both authors (AAMK, JD). Based on the overall quality score, the quality of the included studies could be designated as either poor, moderate or good, where poor specifies total scores ≤ 35 and ≤32 for studies with and without control groups, respectively; moderate specifies total scores between >35 and ≤45 for studies with control groups and scores from >32 to ≤40 for studies without control groups; and good specifies total scores > 45 for studies with control groups and scores >40 for studies without control groups [83]. Disagreements between the authors on the rating of individual items were identified and solved during a consensus discussion. Thorough instructions of the Q-Genie tool are described elsewhere [84]. An overview of the selected genetic association studies is presented in Table 2.

### 3.3. Data Extraction

Removal of duplicated articles was followed by screening of abstracts in order to identify the eligible publications. All steps of the data extraction process were performed independently by both of the reviewers (AAMK, JD).

## 4. Conclusions

In conclusion, studies analyzing the taste-related genetic background of various alcohol consumption behaviors have mainly been carried out on rs713598, rs1726866 and rs10246939 polymorphisms of the TAS2R38 gene encoding for the taste receptor 2 member 38 protein. Due to the inconclusive findings on these variants and to the very limited number of studies on polymorphisms previously linked to sweet taste preference, and on other genetic variants as well, future studies with careful phenotype harmonization are essential to clarify the effect of these polymorphisms. Thus, additional extensive research is recommended to replicate the existing findings. Furthermore, investigating the underlying molecular basis and mechanism of the effect of these polymorphisms on drinking habits is essential to generate new knowledge on the relationship between various taste preference gene polymorphisms and the intake of various types of alcoholic drinks and alcohol consumption phenotypes.

The findings may enhance our understanding of the complexity of alcohol consumption behavior and will aid in the development of personalized recommendations to address different patterns of alcohol consumption behavior such as hazardous and harmful alcohol use, as well as possible alcohol dependence.

## Figures and Tables

**Table 1:** Description of single nucleotide polymorphisms included in the review.

Gene/Nearest Gene	Encoded Protein	SNP ID	Alleles (dbSNP)	Effect on Taste Perception/Preference (if Available)
TAS2R38	Taste 2 Receptor Member 38	rs713598, rs1726866, rs10246939	C > A, G, T G > A T > C	rs713598 is the first location (P/A), rs1726866 is the second location (A/V) and rs1024693 is the third location (V/I) when considering haplotypes [23]. PAV (proline–alanine–valine) homozygotes (dominant haplotype) define the taster form, AVI (alanine–valine–isoleucine) homozygotes the non-taster phenotype and heterozygotes are characterized by intermediate sensitivity to PROP and PTC [21]. rs713598 has the greatest effect on bitter taste signal transduction, rs1726866 possesses weaker effects and rs10246939 site has no detectable effect at all [24].
TAS1R2	Taste 1 Receptor Member 2	rs35874116	T > C	CC and CT vs. TT was found to be associated with higher intake of sweet foods [21]. This SNP (single nucleotide polymorphisms) is located in the primary extracellular domain of the T1R2, which is predicted to harbor the ligand-docking site for carbohydrates and sweet-tasting molecules [25], would contribute to differences in sweet taste perception and food and wine intake [26].
TAS1R3	Taste 1 Receptor Member 3	rs307355	T > A, C	Located in the 5′UTR (untranslated region) of TAS1R3, this cytosine to thymine substitution may affect gene transcription [27] and lead to changes in sweetness and alcohol perception [26]. Reduced taste sensitivity to sucrose was also identified in case of the T alleles of this variant [21].
TAS2R10	Taste 2 Receptor Member 10	rs10845219	T > C	-
TAS2R10 [28]	Taste 2 Receptor Member 10	rs4763216	C > A, G	-
TAS2R13	Taste 2 Receptor Member 13	rs1015443	T >A, C	No association with bitterness of capsaicin, piperine, ethanol and PROP [29,30,31,32]. Minor allele may negatively influence the functioning of TAS2R13 [28].
TAS2R14	Taste 2 Receptor Member 14	rs7138535	T > A	No association with stevioside perception [33].
TAS2R16	Taste 2 Receptor Member 16	rs846664	A > C, G	The derived allele of this nonsynonymous mutation has been shown to increase sensitivity to toxic b-glucopyranosides [34] and was linked to higher sensitivity to salicin but not to PROP bitterness [21]. This variation leads to a functional change in the receptor [35]. The substitution is located in the extracellular loop 2 between transmembrane domains 4 and 5, which are responsible for ligand binding [36,37]. This change may lead to altered taste-related signaling and bitter responsiveness potentially resulting in differences in alcohol preference/consumption.
TAS2R16	Taste 2 Receptor Member 16	rs978739	T > C, G	No association with PROP bitterness [31].
TAS2R16	Taste 2 Receptor Member 16	rs860170	C > T	The A allele was associated with perception of salicin bitterness [31].
TAS2R16	Taste 2 Receptor Member 16	rs1204014	C > G, T	-
TAS2R16 [28,38]	Taste 2 Receptor Member 16	rs1308724	G > A, C, T	No association was found with bitterness of Acesulfame Potassium and quinine [39,40].
TAS2R16 [38,41,42]	Taste 2 Receptor Member 16	rs846672	A > C, T	No association was observed with quinine bitterness [40].
TAS2R19	Taste 2 Receptor Member 19	rs10772420	G > A	Allele A was associated with more intense quinine and grapefruit juice perception [21], which can be due to strong linkage disequilibrium (LD) between TAS2R19 and TAS2R31 SNPs [43].
TAS2R20	Taste 2 Receptor Member 20	rs12226920	G > A, T	Associated with bitter grosheimin [44] and quinine [40] intensities.
TAS2R20	Taste 2 Receptor Member 20	rs10845281	T > A, C, G	Associated with bitter grosheimin intensities [44].
TAS2R3	Taste 2 Receptor Member 3	rs765007	T > A, C	Located in the 5′UTR; therefore may regulate translation efficiency or messenger RNA stability [38]. No association with bitterness of capsaicin, piperine, ethanol threshold [45] and coffee liking, though TAS2R3, -R4, and -R5 haplotype allelic variations explained variability in coffee bitterness (individuals with 1 or 2 copies of the more responsive haplotype (TGAG) experienced twice as much bitterness compared with individuals homozygous for the less-responsive haplotype (CCGT) [38].
TAS2R39	Taste 2 Receptor Member 39	rs4726600	G > A, C	No association was identified with quinine bitterness [40].
TAS2R4	Taste 2 Receptor Member 4	rs2233998	T > C	This non-synonymous SNP was predicted by SIFT algorithm to alter function [46]. No associations were found with PROP, bitterness of capsaicin, piperine, ethanol [29,30,31].
TAS2R4	Taste 2 Receptor Member 4	rs2234001	G > A, C, T	Research suggests that TAS2R3 rs2270009 alters 5 transcription factor binding motifs and consecutive alterations in the secondary structure and stability of T2R3 with the concomitant expression of rs2234001 C–T2R4 may cause altered ligand sensing [47]. No associations were found with Intensity ratings (test samples: sucrose, gentiobiose, aspartame and rebaudioside A and D) [39].
TAS2R40	Taste 2 Receptor Member 40	rs10260248	C > A	-
TAS2R40 [28,48]	Taste 2 Receptor Member 40	rs534126	C > G, T	-
TAS2R41	Taste 2 Receptor Member 41	rs12666496	A > T	-
TAS2R41	Taste 2 Receptor Member 41	rs1404635	G > A, C	-
TAS2R43	Taste 2 Receptor Member 43	rs71443637	T > C	Associated with grosheimin detection threshold and intensities [44] and coffee liking [45].
TAS2R46	Taste 2 Receptor Member 46	rs2708377	C > A, G, T	It is located just adjacent to the coding region of the gene [49], whose receptor is activated by caffeine in vitro [50]. Associated with the perceived bitterness and detection threshold of caffeine [49].
TAS2R5	Taste 2 Receptor Member 5	rs2227264	G > A, T	This non-synonymous SNP was predicted by SIFT algorithm to alter function [46].
TAS2R5	Taste 2 Receptor Member 5	rs2234012	A > G	Located in the 5′UTR; therefore may regulate translation efficiency or messenger RNA stability [38].
TAS2R50	Taste 2 Receptor Member 50	rs1376251	C > T	C allele was associated with dietary fiber and vegetable intake [42]. Also associated with bitter-tasting grosheimin strong, very strong intensity [44].
TAS2R50	Taste 2 Receptor Member 50	rs10772397	C > A, G, T	Analyzed, but no association was found with bitterness of quinine [40].
TAS2R60	Taste 2 Receptor Member 60	rs4595035	T > A, C, G	Being a synonymous polymorphism [38], it was hypothesized that it may have an altered function. Analyzed, but no association was found with quinine bitterness [40].
TAS2R7	Taste 2 Receptor Member 7	rs619381	C > T	May impact TAS2R expression (it affects an amino acid in the C-terminal domain of TAS2R7, thus unlikely to directly impact ligand interactions) [15].
TAS2R8	Taste 2 Receptor Member 8	rs1548803	C > T	Associated with quinine intensity [40].
CA6	Carbonic Anhydrase 6	rs2274333	A > G	In some studies A alleles associated with supertasting of PROP [21].
GNAT3	G Protein Subunit Alpha Transducin 3	rs1524600	G > A	C alleles associated with higher sensitivity for sucrose [51].
PRH1 *	Proline Rich Protein HaeIII Subfamily 1	rs10492098	G > A, C	Salivary levels of some peptides of the proline rich protein family have been suggested to modulate bitter taste perception [52].
PRH1-TAS2R14	Proline Rich Protein HaeIII Subfamily 1—Taste 2 Receptor Member 14	rs2597979	G >A, C, T	G allele associated with increased intensity rating of PROP [53].
PRH1-TAS2R14	Proline Rich Protein HaeIII Subfamily 1—Taste 2 Receptor Member 14	rs11612527	T > A	-
PRR4 *	Proline Rich 4	rs1047699	T > A, C	PRR4 may have an association to Lipocalin-1 (LCN1), which could play a role in taste reception [54].
PRR4 *	Proline Rich 4	rs1063193	C > A, G, T	PRR4 may have an association to Lipocalin-1 (LCN1), which could play a role in taste reception [54].
TRPA1	Transient Receptor Potential Cation Channel Subfamily A Member 1	rs11988795	C > A, G, T	Investigated, but no association was found with quinine [40]. Allele A was associated with enhanced perception of odorous stimulants [55].

Italic gene names refer to nearest genes as used by cited authors. * Potential role in taste perception according to the STRING Database (https://string-db.org, accessed on 22 November 2022). No association or no explanation has yet been found/investigated for the potential effect of the variant.

**Table 2:** Overview of the genetic association studies included in the systematic review.

Publication (First Author, Year)	Gene	SNP	Study Population Characteristics	Phenotype Assessment Method	Findings
Beckett (2017) [56]	TAS2R38	rs713598	180 Australian hospital patients (51% females; mean age 61.6 years).	FFQ	TAS2R38 rs713598 “P” allele carriers consumed fewer standard drinks per day and fewer standard drinks per day from spirits, and from mixed drinks compared to those without P allele.
Hayes (2011) [38]	TAS2R16	rs846672	96 healthy adults from Connecticut (mean age 40.9 years, 76% females).	Semiquantitative food frequency survey	Individuals with the AA genotype of TAS2R16 rs846672 consumed alcoholic beverages twice as frequently and the quantity of alcohol consumed was more than in case of people with other genotypes.
	TAS2R38	rs713598, rs1726866, rs10246939			TAS2R38 AVI homozygotes drank more than either heterozygotes or PAV homozygotes, and AVI homozygotes consumed alcohol more frequently than PAV homozygotes.
	TAS2R16	rs1308724			CC homozygotes of TAS2R16 rs1308724 consumed alcohol less frequently than heterozygotes, who also consumed less frequently than GG homozygotes.
Ramos-Lopez (2015) [57]	TAS2R38	rs713598, rs1726866, rs10246939	375 individuals (182 females, 193 males) from the city of Guadalajara (state of Jalisco, Mexico).	Medical history questionnaire	In comparison with heterozygotes and PAI homozygotes, TAS2R38 AVV/AVV genotype was significantly associated with alcohol intake, and the frequency of AVV homozygotes was significantly higher among drinkers than nondrinkers.
Duffy (2004) [58]	TAS2R38	rs713598, rs1726866, rs10246939	84 healthy adults (53 women and 31 men; 21–59 years).	Block Food Survey	TAS2R38 AVI/AVI homozygotes drank significantly more alcohol than either the PAV/AVI heterozygotes or the PAV/PAV homozygotes.
Fu D. (2019) [41]	TAS2R38	rs10246939, rs1726866, rs713598	519 respondents (52% females; 21 years of age or older) in California.	Alcohol consumption frequency	TAS2R38 (rs10246939, rs1726866, rs713598) showed significant association with alcohol consumption, and individuals carrying alleles allowing perception of bitterness in PTC consumed more alcohol.
	CA6	rs2274333			
	GNAT3	rs1524600			
	TAS2R16	rs846664,			
	TAS2R19	rs10772420			
	TAS2R20	rs12226920			
	TAS2R43	rs71443637			
	TAS2R46	rs2708377			
	TAS2R50	rs10772397			
	TAS2R60	rs4595035			
	TAS2R8	rs1548803			
	TRPA1	rs11988795			
	TAS2R16	rs846672			
Hinrichs (2006) [35]	TAS2R16	rs846664, rs978739, rs860170, rs1204014	A total of 262 families (2310 individuals).	Alcohol dependence	Individuals with the ancestral allele of TAS2R16 rs846664 were found to be at increased risk of alcohol dependence, regardless of ethnicity.
Wang (2007) [59]	TAS2R38	rs713598, rs1726866, rs10246939	262 families (2309 individuals).	SSAGA	The common taster haplotype TAS2R38 was significantly associated with a lower mean of the largest number of drinks that participants had ever had in a 24 h period compared with the other haplotypes.
	TAS2R16	rs846664			The TAS2R16 rs846664 allele associated with lower alcohol dependence risk and with lower mean of the largest number of drinks that participants had ever had in a 24 h period.
Choi (2016) [60]	TAS2R38	rs713598, rs1726866, rs10246939	1524 Korean participants (males 832, females 748).	FFQ	TAS2R38 diplotype was not associated with alcohol consumption (g/day).
Choi (2017) [26]	TAS2R38	rs713598, rs1726866, rs10246939	1829 participants (males 997, females 832).	Questionnaire	Participants with TAS2R38 AVI haplotype were less likely to be a drinker while TAS2R5 rs2227264 TT consumed more alcohol than other genotypes. TAS1R3 rs307355 CT carriers were associated with heavy drinking status and choice of alcoholic beverages. The homo-recessive type of TAS2R4 rs2233998 and TAS2R5 rs2227264 were associated with consumption of rice wine while TAS1R2 rs35874116C allele carriers were less likely to drink wine, and CC subjects had higher levels of intake than other genotypes. TAS2R50 rs1376251 CC drank more rice wine and spirits. TAS2R20 rs12226920 showed an association with the consumption of spirits.
	TAS2R5	rs2227264			
	TAS2R4	rs2233998			
	TAS1R3	rs307355			
	TAS1R2	rs35874116			
	TAS2R50	rs1376251			
	TAS2R20	rs12226920			
Keller (2013) [61]	TAS2R38	rs713598, rs1726866, rs10246939	1007 subjects (405 males, 602 females; mean age 48) from Eastern Germany.	Questionnaire	Categorizing individuals carrying at least one TAS2R38 PAV haplotype vs. AVI/AVI homozygous carriers, lower alcohol intake per week was observed in the PAV group.
Vinuthalakshmi (2019) [62]	TAS2R38	rs713598, rs1726866, rs10246939	296 subjects of Koraga primitive tribes (Southwest coast of Karnataka and Kerala state of India).	Questionnaire	Positive significant association was found between the TAS2R38 AVI/AVI haplotype and alcoholics.
Dotson (2012) [28]	TAS2R38	rs713598 rs10246939 rs1726866	173 (126 men and 47 women; mean age 60.7) head and neck cancer patients from clinics at the University of Florida.	The first three questions of the AUDIT	TAS2R38 was significantly associated with the first question of the AUDIT screening tool. The major allele, C rs713598 was strongly associated with decreased alcohol consumption. rs1015443 of TAS2R13 was significantly associated with the second and third questions of the AUDIT screening tool. CC homozygotes consumed alcoholic beverages less frequently compared to heterozygotes and minor allele homozygotes.
	TAS2R13	rs1015443			
	TAS2R3	rs765007			
	TAS2R4	rs2234001			
	TAS2R5	rs2234012			
	TAS2R7	rs619381			
	TAS2R8	rs1548803			
	TAS2R10	rs10845219			
	TAS2R14	rs7138535 rs1376251			
	TAS2R19	rs10772420			
	TAS2R20	rs10845281			
	TAS2R39	rs4726600			
	TAS2R40	rs10260248			
	TAS2R41	rs1404635			
	TAS2R10	rs4763216			
	PRH1-TAS2R14	rs11612527			
	EPHA1-AS1	rs12666496			
	PRH1	rs10492098			
	PRR4	rs1047699 rs1063193			
	TAS2R16	rs1308724 rs846672			
	TAS2R40	rs534126			
Choi (2019) [63]	TAS2R38	rs713598, rs1726866, rs10246939	3567 participants (1338 males and 2229 females; ages from 40 to 89 years) from three rural areas (Goryeong, Namwon, Yangpyeong) of Korea.	Questionnaire	No association of TAS2R38 with alcohol intake.
Choi (2017) [65]	TAS2R38	rs713598, rs1726866, rs10246939	2042 subjects (males 1390, females 652; mean age 56.1), from Korea.	Questionnaire	Either the individual or combined effect of TAS2R38 and CA6 genetic variants had no influence on daily alcohol consumption.
	CA6	rs2274333			
Timpson (2005) [64]	TAS2R38	rs713598, rs1726866, rs10246939	3383 British women (aged 60 to 79).	Questionnaire	No substantial evidence of significant association between TAS2R38 PAV and AVI haplotypes and alcohol consumption.
Schembre (2013) [42]	TAS2R38	rs713598, rs1726866, rs10246939	914 colorectal adenoma cases (males 60.2%) and 1188 controls (males 62.7%) (mean age of 60.6) years.	FFQ	No significant associations were identified between the TAS2R38 PAV/PAV diplotype, TAS2R50 rs1376251 and the TAS2R16 rs846672 polymorphisms with alcohol intake (mg/day).
	TAS2R50	rs1376251			
	TAS2R16	rs846672			
Ong (2018) [53]	TAS2R38	rs1726866	438 870 participants (males 45.8%; mean age: 56.5) of England, Wales and Scotland.	Drinking behavior	TAS2R38 rs1726866 was inversely associated with alcohol consumption.
	TAS2R19	rs10772420			
	PRH1-TAS2R14	rs2597979			

Italic gene names refer to nearest genes as used by cited authors.

**Table 3:** Overview of the effect of taste preference-related gene polymorphisms on alcohol consumption.

Gene	SNP	Association	No Association
TAS2R38	rs713598, rs1726866, rs10246939	AVI/AVI homozygotes drank alcohol more frequently and consumed significantly more alcoholic beverages than other genotypes [38]. Frequency of AVV homozygotes was significantly higher among drinkers when compared to non-drinkers and was associated with increased alcohol intake when compared with heterozygotes and PAI homozygotes [57]. AVI/AVI homozygotes consumed significantly more alcoholic beverages than other genotypes [58]. The common taster haplotype was significantly associated with a lower mean of the largest number of drinks that participants ever had in a 24 h period compared with the other haplotypes [59]. The dominant model analyses (PAV/PAV vs. PAV/AVI+AVI/AVI) confirmed that the subjects with the AVI haplotype were less likely to be a drinker [26]. Positive association between non-taster haplotype AVI/AVI and being alcoholic [62]. Lower alcohol intake (per week) was observed among subjects with at least one PAV haplotype vs. AVI/AVI homozygous subjects [61]. A significant association was found with alcohol consumption frequency. Carriers of the allele allowing perception of bitterness in PTC consumed alcohol at higher frequencies [41].	No association with alcohol consumption. TAS2R38 diplotypes (PAV/PAV, PAV/AVI and AVI/AVI) showed no significant differences in daily alcohol consumption [60]. No association with beer consumption and total daily alcohol consumption [26]. No association with alcohol drinker status [63]. No association with daily consumption of alcohol (g/day) [65]. No association with drinker status [64]. No association with frequency and amount of alcohol consumed [42].
TAS2R38	rs713598	The variant was a significant predictor of the number of standard drinks consumed. Subjects carrying the p allele consumed fewer standard drinks per day and fewer standard drinks per day from spirits, and from mixed drinks, compared non-carriers [26].	No association with beer and wine consumption [26].
		A significant association was found with alcohol consumption frequency. Carriers of the allele allowing perception of bitterness in PTC consumed alcohol at higher frequencies [30].	No significant association with the second and third questions of the AUDIT screening tool [37].
		The C allele of this variant was strongly associated with decreased alcohol consumption measured by the first question of the AUDIT screening tool in a cohort of head and neck cancer patients [37].	No association with alcohol intake per week [35].
TAS2R38	rs1726866	Inverse significant association with alcohol consumption frequency and drinker status [42].	No significant association with the first three questions of the AUDIT screening tool [37].
		A significant association with alcohol consumption frequency was found. Carriers of the allele allowing perception of bitterness in PTC consumed alcohol at higher frequencies [30].	No association with alcohol intake per week [35].
TAS2R38	rs10246939	A significant association with alcohol consumption frequency was found. Carriers of the allele allowing perception of bitterness in PTC consumed alcohol at higher frequencies [41].	No association with alcohol intake per week [61]. No significant association with the first three questions of the AUDIT screening tool [28].
TAS1R2	rs35874116	The variant is responsible for both wine consumption status and intake. Participants with the C allele were less likely to be wine drinkers. Moreover, subjects with the CC recessive type exhibited higher levels of wine intake compared to the other genotypes [26].	No significant association with Soju, beer and spirit consumption [26].
TAS1R3	rs307355	Drinkers of the heterozygous genotype were more likely to be heavy drinkers (≥30 g/day) than those with the wild genotype. Exhibited a marginal association with total Soju intake [26].	No significant association with beer, wine and spirit consumption [26].
TAS2R10	rs10845219		No significant association with the first three questions of the AUDIT screening tool [28].
TAS2R10	rs4763216		No significant association with the first three questions of the AUDIT screening tool [28].
TAS2R13	rs1015443	Significantly associated with the second and third questions of the AUDIT screening tool. Participants with homozygous major allele (CC carriers) consumed alcoholic beverages less frequently compared to heterozygotes as well as homozygous for the minor allele [28].	
TAS2R14	rs7138535		No significant association with the first three questions of the AUDIT screening tool [28].
TAS2R16	rs846664	Ancestral allele K172 was associated with increased risk of alcohol dependence, regardless of ethnicity [35]. The allele associated with lower alcohol dependence risk was also associated with lower mean of the largest number of drinks that participants had ever had in a 24 h period [42].	No significant association with frequency of alcohol consumption [41].
TAS2R16	rs846672	AA carriers consumed alcoholic beverages twice as frequently as the heterozygotes or major allele homozygotes and also drank more than G allele carriers [38].	No association with frequency and amount of alcohol consumed [42]. No significant association with frequency of alcohol consumption [41]. No significant association with the first three questions of the AUDIT screening tool [28].
TAS2R16	rs1308724	CC homozygotes consumed alcohol less frequently than heterozygotes, who also consumed less frequently than GG homozygotes [38].	No significant findings for total intake of alcohol [38]. No significant association with the first three questions of the AUDIT screening tool [28].
TAS2R16	rs978739		No association with alcohol dependence [35].
TAS2R16	rs860170		No association with alcohol dependence [35].
TAS2R16	rs1204014		No association with alcohol dependence [35].
TAS2R19	rs10772420		No significant association with frequency of alcohol consumption [41]. No significant association with the first three questions of the AUDIT screening tool [28]. No significant association with drinking behavior frequency and heavy drinker status [53].
TAS2R20	rs12226920	An association was found with drinker status of spirits (but not with logistic regression models) [26].	No association with beer consumption [41].
TAS2R20	rs10845281		No significant association with the first three questions of the AUDIT screening tool [28].
TAS2R3	rs765007		No significant association with the first three questions of the AUDIT screening tool [28].
TAS2R39	rs4726600		No significant association with the first three questions of the AUDIT screening tool [28].
TAS2R4	rs2233998	The subjects with the homo-recessive type of this polymorphism were more likely to be rice wine drinkers compared to other genotypes [26].	No significant association with Soju, beer and spirit consumption [26].
TAS2R4	rs2234001		No significant association with the first three questions of the AUDIT screening tool [28].
TAS2R40	rs10260248		No significant association with the first three questions of the AUDIT screening tool [28].
TAS2R40	rs534126		No significant association with the first three questions of the AUDIT screening tool [28].
TAS2R41	rs12666496		No significant association with the first three questions of the AUDIT screening tool [28].
TAS2R41	rs1404635		No significant association with the first three questions of the AUDIT screening tool [28].
TAS2R43	rs71443637		No significant association with frequency of alcohol consumption [41].
TAS2R46	rs2708377		No significant association with frequency of alcohol consumption [41].
TAS2R5	rs2227264	An association was found with the level of total alcohol intake. TT genotype individuals consumed more alcohol and were more likely to be rice wine drinkers than those with other genotypes [26].	No significant association with Soju, beer and spirit consumption [26].
TAS2R5	rs2234012		No significant association with the first three questions of the AUDIT screening tool [28].
TAS2R50	rs1376251	An association was found with drinker status of spirits (but not with logistic regression models). Moreover, participants with the CC genotype tended to drink more rice wine when compared to other genotypes [26].	No association with daily alcohol intake (mg/day) [42]. No significant association with Soju, beer and wine consumption [26]. No significant association with the first three questions of the AUDIT screening tool [28].
TAS2R50	rs10772397		No significant association with frequency of alcohol consumption [41].
TAS2R60	rs4595035		No association with the frequency of alcohol consumption [41].
TAS2R7	rs619381		No significant association with the first three questions of the AUDIT screening tool [28].
TAS2R8	rs1548803		No significant association with the first three questions of the AUDIT screening tool [28]. No significant association with frequency of alcohol consumption [41].
TRPA1	rs11988795		No significant association with frequency of alcohol consumption [41].
CA6	rs2274333		No association with daily consumption of alcohol (g/day) [65]. No significant association with frequency of alcohol consumption [41].
GNAT3	rs1524600		No statistically significant association with frequency of alcohol consumption [41].
PRH1	rs10492098		No significant association with the first three questions of the AUDIT screening tool [28].
PRH1-TAS2R14	rs2597979		No significant association with drinking behavior frequency and heavy drinker status [53].
PRH1-TAS2R14	rs11612527		No significant association with the first three questions of the AUDIT screening tool [28].
PRR4	rs1047699		No significant association with the first three questions of the AUDIT screening tool [28].
PRR4	rs1063193		No significant association with the first three questions of the AUDIT screening tool [28].

Italic gene names refer to nearest genes as used by cited authors.

## Data Availability

Not applicable.
